# Public Commencement of Rush Medical College

**Published:** 1856-02

**Authors:** 


*Public Commencement of Rush Medical College.*

The closing exercises of the Thirteenth Annual Session of Rush Medical College in this city, took place on Wednesday evening, the 20th of February. The Metropolitan Hall was well filled with an attentive audience. The Degree of Doctor of Medicine was conferred on 42 members of the class; the *ad-eundem* degree, on Dr. JOSEPH HENDERSON, of Knox Co.,

Ill.; and the Honorary Degree, on Dr. M. M. LATTA, of Goshen, Indiana. An interesting valedictory address was delivered by Prof. W. B. HERRICK. His subject was the History of Medicine, and was listened to with marked attention. After the address, the graduates, students, alumni, and invited guests repaired to the Briggs House, and partook of an elegant collation, accompanied by a season of high social enjoyment, which will not be soon forgotten. The whole number of Matriculants, during the college term just closed, was 150. The following are the names of the graduating class:—

John E. Deming, North Oxford, Mich.
Samuel Griffith, Moundsville, Ill.
Robert Hitt, Mt. Morris, Ill.
Hamilton C. Daniels, Naperville, Ill.
Daniel McM. Marshall, Hopper's Mills, Ill.
Joseph M. Williamson, Lovington, Ill.
Amzi B. Cary, Greenbush, Wis.
Alexander A. Lodge, Hitesville, Ind.
Meredith C. Archer, Young, Wis.
J. Milton Barlow, Bell Air, Ill.
John R. Robson, New Harmony, Ind.
Francis Ronalds, Evansville, Ind.
Horace Wardner, Chicago, Ill.
Thos. C. McGee, Winchester, Va.
Robert Winton, Wheeling, Va.
Edwin Gaylord, Magnolia, Ill.
William H. Philips, Kenton, Ohio.
James P. Graham, Clermont, Ind.
William F. Green, Shelbyville, Ind.
James W. Green, Burlington, Iowa.
James L. Crain, Urbana, Ill.
A. Jackson Crain, Urbana, Ill.
Zephaniah H. Madden, Clinton, Ill.
Lucien L. Leeds, Lincoln, Ill.
Benj. G. Neal, Columbus City, Ia.
Benj. S. Lewis, Marion, Ill.
David La Count, Manitowoc, Wis.
William A. Gordon, Hanson, Wis.
Edward W. Boothe, Vandalia, Ill.
David T. Kyner, Springfield, Ill.
Francis M. Constant, Mexico, Ind.
Roswell Eaton, Delavan, Wis.
Daniel Bowers, Hardensville, Ill.

John J. Everhard, LaCote St. Marie, Wis.
George W. Kittell, Sherbon, Ill.
John W. F. Clawges, La Harp, Ill.
Henry W. Kreider, Fairview, Ill.
Bailey Ragon, Green Bush, Ill.
David W. Carley, Shullsburg, Wis.
Lee Smith, Hudson, Ill.
Almon C. Buffum, Chicago, Ill.

