# Errors

**Published:** 1856-03

**Authors:** 


ERRORS.—In the list of graduates published in the February number of the Journal, the name of J. N. Higgins, of Griggsville, Ill., was accidentally omitted.

The address of Robert Winton was given "Wheeling, Va.," which should have been Wheeling, Ind.

